# Supplementary material for: Association between oral microbiome and seven types of cancers in East Asian population: a two-sample Mendelian randomization analysis
Source: Front Mol Biosci. 2023 Nov 21;10:1327893. doi: 10.3389/fmolb.2023.1327893 (PMC10702768; doi:10.3389/fmolb.2023.1327893)
Supplement: Supplementary file 1 [file DataSheet1.ZIP › Supplementary Materials/MR plots of saliva/saliva═╝/Lung Cancer/pheno.43_to_lung cancer_forest.pdf]

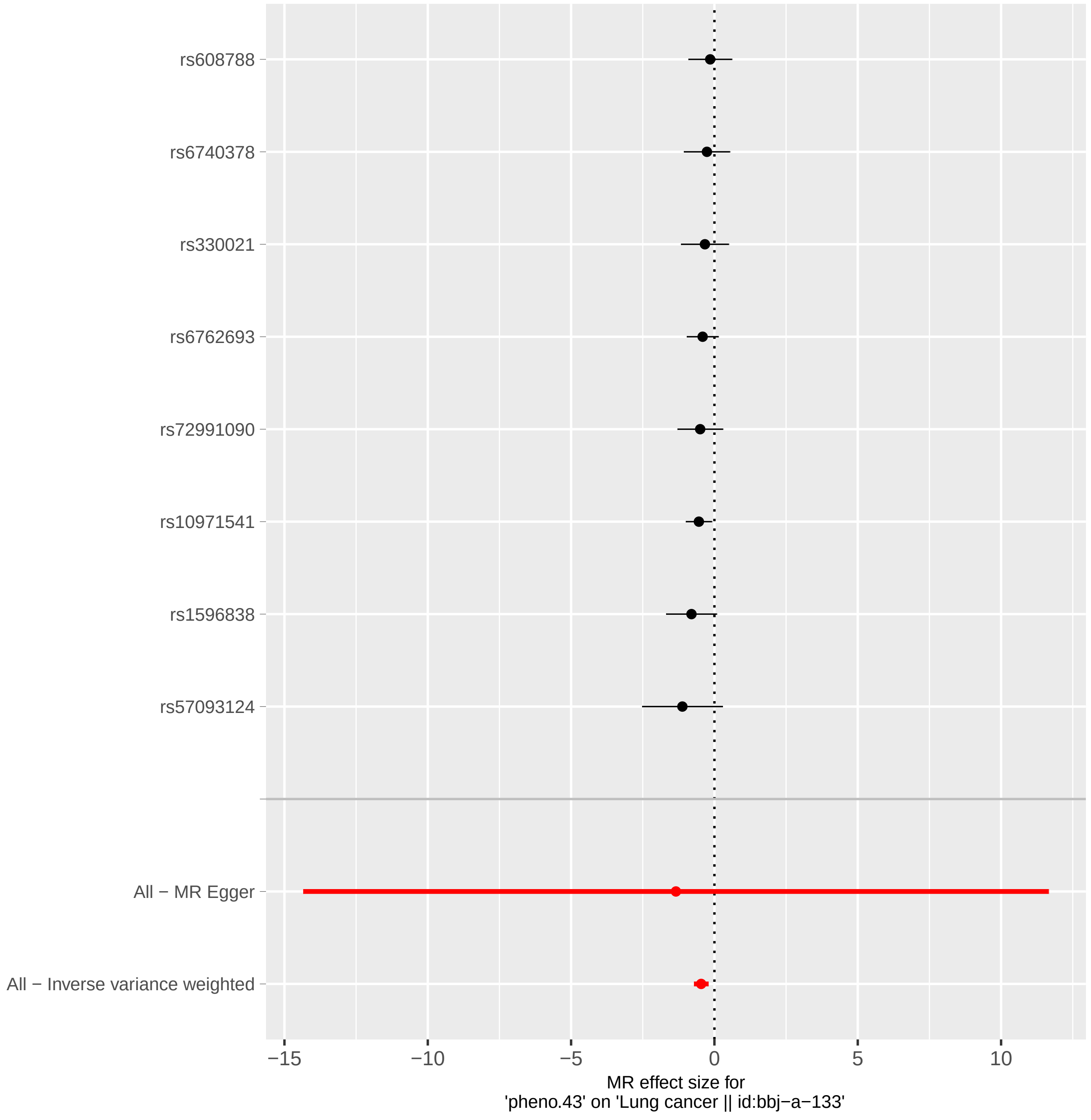

rs608788
rs6740378
rs330021
rs6762693
rs72991090
rs10971541
rs1596838
rs57093124
All − MR Egger
All − Inverse variance weighted
−15
−10
−5
0
5
10
MR effect size for
'pheno.43' on 'Lung cancer || id:bbj−a−133'
